# American Journal of Ophthalmology

**Published:** 1861-12

**Authors:** 


"*American Journal of Ophthalmology.*"—Dr. Homberger, a valued though but recent contributor to the pages of the *Monthly*, requests us to announce his design to publish a journal entitled as above, especially devoted to Ophthalmic Medicine and Surgery. To speak of the advantages which such a publication offers to the specialist as well as to the general practitioner seems almost superfluous. There was a time when all natural science could be grasped by one master-mind. As the knowledge of principles and facts accumulated, divisions and sub-divisions were found necessary. Thus medicine, as a speciality, became separate from its fountain-head and co-ordinate streams, the physical sciences. Of late years, our science and art has received such an impetus by men of genius, and enlarged general medical education, having devoted themselves to special departments of study or practice exclusively, that it has become as impracticable to represent, without a division of labor, the actual advances in the whole domain of medicine by the periodical press, as it is impossible for one man to teach all the branches in our schools, or to attain pre-eminence in every branch in actual practice. We shall recur to this subject at a future time; meanwhile, we bespeak for the proposed Journal of a speciality as emancipated from general practice as that of the oculist has become, a favorable consideration. [*American Medical Monthly*.

